# Medical Expulsive Therapy for Distal Ureteral Stones: Tamsulosin Versus Silodosin in the Turkish Population

**DOI:** 10.7759/cureus.1848

**Published:** 2017-11-15

**Authors:** Ersan Arda, Basri Cakiroglu, Ilkan Yuksel, Esra Akdeniz, Gizem Cetin

**Affiliations:** 1 Urology, Trakya University Medical Faculty; 2 Urology, Hisar Intercontinental Hospital; 3 Urology, Private Bir Nefes Hospital; 4 Biostatistics, Marmara University School of Medicine; 5 Anesthesiology, Trakya University Medical Faculty

**Keywords:** tamsulosin, silodosin, ureter, stone

## Abstract

Introduction

Our aim was to contribute a study that includes a higher patient population to the limited number of studies comparing tamsulosin and silodosin in the treatment of distal ureteral stones.

Material and methods

Patients who presented with renal colic to the urology emergency clinic and were diagnosed with ureteral stones and followed-up with conservative treatment between January 2010 and January 2016 were retrospectively screened. According to the inclusion-exclusion criteria, the patients were divided into three groups. Group 1: 150 patients followed with watchful waiting (WW), Group 2: 156 patients who received 0.4 mg of tamsulosin daily, and Group 3: 159 patients who received 8 mg of silodosin daily. The side effects of the used drugs, duration of stone reduction, and expulsion rates were evaluated and compared separately.

Results

A total of 465 patients were included in the study. No statistically significant difference was found in terms of age, gender, and stone size among the groups. The patient characteristics and results are shown in Table 1. The differences in stone expulsion rate between the groups in the first week were calculated using the Chi-square test and found to be non-significant (p = 0.155); whereas, the stone expulsion rates between Group 1 versus Group 2 and Group 1 versus Group 3 were found to be significantly different after the second and third week.

Conclusion

According to our results, no statistically significant superiority between tamsulosin and silodosin was shown in the treatment of distal ureteral stones in the Turkish population.

## Introduction

Urinary system stone disease is the third most common urological disorder after urinary infections and prostate diseases [1]. Twenty percent of the detected stones are ureteral, and nearly 70% of them are in the distal 1/3 part of the ureter [2].

Conservative methods such as watchful waiting (WW) or medical expulsive therapy (MET) have been increasingly widespread latterly in the treatment of distal ureteral stones for spontaneous stone passage, especially in patients until grade 2 hydronephrosis [3].

The presence of three alpha 1 receptor subtypes (alpha 1a, 1b, and 1d) in the human ureter have been shown. Alpha 1a and 1d antagonists have been proven to increase the probability of stone expulsion due to the decreased baseline ureteral tone, peristaltic contraction, intraluminal pressure, and increased urine flow [4]. Thus, without the need for additional intervention, MET, which includes alpha blocker agents, has become popular in order to increase spontaneous stone expulsion rate, shorten the dropping period, and reduce the need for pain relief [5].

Tamsulosin, which has high uroselectivity and shows equal alpha 1a and 1d activity, is widely used in studies and has proven its role in MET for a long time [6-8]. Recently, it has been suggested that silodosin, which is higher alpha 1a selective and nowadays increasingly frequently used, is more potent in MET compared to tamsulosin [9-11]. To date, however, there are scarce and controversial clinical studies in the medical treatment of ureteral stones.

Our aim was to evaluate conservative treatment approaches for distal ureteral stones by comparing WW, tamsulosin, and silodosin and to contribute a study that includes a higher patient population from Turkey to the limited literature.

## Materials and methods

Between January 2010 and January 2016, patients who presented with renal colic to the urology emergency clinic, were diagnosed with ureteral stones, and followed-up with conservative treatment were retrospectively screened. The data of 960 patients were obtained on radiographic images. The criteria for inclusion in the study were stone diameters below 1 cm and above 3 mm, unilaterality, radiodensity, and distal ureteral presence. The exclusion criteria were solitary kidney, grade 3 and/or 4 hydronephrosis, and previous renal or ureteral surgery.

A total of 465 patients who met the criteria were included in the study and divided into three groups. Group 1: 150 patients followed with WW, Group 2: 156 patients who received 0.4 mg of tamsulosin daily, and Group 3: 159 patients who received 8 mg of silodosin daily. Patient follow-up was done once a week for three weeks with routine radiographic and ultrasonic assessment to compare stone expulsion rates. During this survey, the frequency of analgesic usage was recorded to evaluate pain ratings. A comparison of adverse effects was made between retrograde ejaculation, orthostatic hypotension, dizziness, and headache. Eventually, side effects of the used drugs, duration of stone reduction, and expulsion rates were evaluated and compared separately.

## Results

No statistically significant difference was found in terms of age, gender, and stone size among the groups. The patient characteristics and results are shown in Table 1.

**Table 1:** Patients’ characteristics and results according to treatment

	Control (n=150)	Tamsulosin (n=156)	Silodosin (n=159)	p-value
Age (mean±SD)	42.2 ±11.4	44.61±12.03	45.89±12.90	0.781
Gender (male:female)	94:56:00	97:59:00	111:48:00	0.3562
Stone size (mm) (mean±SD)	5.96 ± 1.18	5.93 ± 1.07	5.94±1.23	0.8351
expulsion rate (%) (after two weeks)	37.2% (56/150)	58.3% (91/156)	62.3% (99/159)	<0.001*
expulsion rate (%) (after three weeks)	50.0% (75/150)	72.4% (113/156)	78.6% (125/159)	<0.001*
Weeks to stone expulsion				0.773
One week of treatment	24.6% (37/150)	32.0% (50/156)	36.5% (58/159)	0.155
Two weeks of treatment	12.6% (19/150)	26.3% (41/156)	25.8% (41/159)	0.029*
Three weeks of treatment	12.6% (19/150)	14.1% (22/156)	16.3% (26/159)	0.746

The number of patients who experienced stone expulsion did not show any significant difference with respect to weeks (p = 0.773). One week stone expulsion rate of 24.6% (37 out of 150 patients) was observed in Group 1, 32% (50 out of 156) was observed in Group 2, and 36.5% (58 out of 159 patients) in Group 3. The differences between the groups was calculated using the Chi-square test and found to be nonsignificant (p = 0.155). Two weeks stone expulsion rate of 37.2% (56 out of 150 patients) was observed in Group 1, 58.3% (91 out of 156) was observed in Group 2, and 62.3% (99 out of 159 patients) in Group 3 (Table 2). The stone expulsion rates after two weeks were found to be significantly different between Group 1 vs. Group 2 and Group 1 vs. Group 3. Both Group 2 and 3 show a statistical advantage over Group 1 in terms of stone expulsion after two weeks.

**Table 2:** Post hoc analyses for cumulative expulsion proportions for each week

	Stone expulsion rate after 2 weeks		Stone expulsion rate after 3 weeks	
Group Comparison	Difference	Critical Range	Difference	Critical Range
Group 1 vs. Group 2	0.211*	0.160	0.224*	0.158
Group 1 vs. Group 3	0.251*	0.159	0.286*	0.154
Group 2 vs. Group 3	0.040	0.135	0.062	0.118

When we considered stone expulsion rate in the second week, 12.6% (19 out of 150 patients) was observed in Group 1, 26.3% (41 out of 156 patients) was observed in Group 2, and 25.8 (41 out of 159 patients) was observed in Group 3. The differences between the groups were calculated using the Chi-square test and found to be nonsignificant (p=0.029). This test was followed by the Marascuilo procedure and the results are given in Table 3. The stone expulsion rates in the second week were found to be significantly different between Group 1 vs. Group 2 and Group 1 vs. Group 3. Both Group 2 and 3 showed a statistical advantage over Group 1 in terms of stone expulsion at the second week. Three weeks stone expulsion rate of 50% (75 out of 150 patients) was observed in  Group 1, 72.4% (113 out of 156) was observed in Group 2, and 78.6% (125 out of 159) was observed in Group 3. The differences between the groups were calculated using the Chi-square test and found to be significant (p<0.001). The Marascuilo procedure in Table 3 states that the stone expulsion rate after three weeks was found to be significantly different between Group 1 vs. Group 2 and Group 1 vs. Group 3. Both Group 2 and 3 showed a statistical advantage over Group 1 in terms of stone expulsion after three weeks.

**Table 3:** Post hoc analyses for expulsion proportions within each week

	Week 1		Week 2		Week 3	
Group Comparison	Absolute Difference	Critical Range	Absolute Difference	Critical Range	Absolute Difference	Critical Range
Group 1 vs. Group 2	0.076	0.146	0.135*	0.123	0.013	0.111
Group 1 vs. Group 3	0.121	0.147	0.130*	0.122	0.035	0.114
Group 2 vs. Group 3	0.045	0.131	0.05	0.121	0.022	0.099

No severe complications were recorded in the groups. Adverse effects are shown in Table 4, where no statistically significant difference was found between the drugs.

**Table 4:** Adverse effects observed in patients

	Tamsulosin (n=156)	Silodosin (n=159)	p-value
Orthostatic hypotension	6 (9/156)	6.3 (10/159)	1
Retrograde ejaculation	8 (12/156)	6 (9/159)	0.619
Headache	11 (17/156)	15 (24/159)	0.348

Statistical analysis

Statistical analyses were conducted using the R program (a GNU project that is a language and environment for statistical computing and graphics). The Kruskal-Wallis test was employed for comparison among the groups. Fisher's exact test and Chi-square test were used to compare groups with respect to nominal variables. The Marascuilo procedure was employed to simultaneously test the differences of all pairs of proportions where a statistically significant value was exceeded. A p < 0.05 was considered to indicate statistical significance.

## Discussion

Alpha blockers are widely used in the medical treatment of distal ureteral stones. Especially in the last decade, MET, which includes alpha blocker agents, has become popular in order to increase spontaneous stone expulsion rate, shorten dropping period, and reduce the need for pain relief. According to our results, in the conservative treatment of distal ureteral stones, MET was found to be significantly superior to WW and this supports the literature [9, 12-13].

When stone expulsion rates, the first endpoint of the study, were analyzed, Group 1, Group 2, and Group 3 showed 50%, 72.4%, and 78.6% efficacy, respectively. In all studies that compared tamsulosin and silodosin for MET, higher efficacy in favor of silodosin was shown, similar to our findings [14-20].

Nevertheless, in a study conducted by Imperatore, et al. that included 100 patients, efficacy levels were found to be 82% for tamsulosin and 88% for silodosin. Although these values ​​were higher than ours in a proportional sense, it has been shown that the drug efficacies were not statistically different, which was supported by our findings [14].

According to the results of another placebo-controlled study published in 2016, efficacy rates for placebo, tamsulosin, and silodosin groups were reported as 50%, 76.6%, and 86.2%, respectively [15]. Similar to our results, no statistically significant difference between the drugs with remarkable similarity in terms of the obtained values was shown ​​[17]. However, the other four studies comparing tamsulosin and silodosin suggested that stone expulsion rates in patients receiving silodosin is statistically higher [17-20].

In the literature, efficacy levels of tamsulosin alone for MET in distal ureteral stones were reported to be over 70% [2,7-8]. However, in these four comparative studies, tamsulosin activity was reported to be 58%, 64.4%, 61.2%, and 57%, respectively [17-20]. It is noteworthy that the success rates were much lower when the published ratios of spontaneous stone-free studies and the publications on tamsulosin alone were taken into consideration. Under these circumstances we thought that the number of patients in these studies were not high enough, which can be an important and decisive factor that led to the statistical superiority.

When stone dropping time, our second target (2nd endpoint) was examined, results for WW, tamsulosin, and silodosin were 24.6%, 32%, and 36.5% in the first week; 12.6%, 26.3%, and 25.8% in the second week; and 12.6%, 14.1%, and 16.3% in the third week, respectively. Stone dropping time was found to be statistically significantly shorter in the drug group compared to WW. When we considered the two studies that showed similiar results in terms of expulsion efficacy to ours, no statistically significant difference was found in terms of expulsion time between the two drugs, which was also supported by our study [14,16].

On the other hand, in a majority of the studies suggesting that silodosin activity is higher, statistically significant results for stone expulsion time have been reported in favor of silodosin too [17-18,20]. However, in a study by Kumar, et al., which had the highest number of patients and tamsulosin activity (64.4%), no statistically significant difference was shown in stone expulsion times, similar to our results [19]. This supports our previously mentioned idea of ​​'patients paucity', which is thought to be the reason for the emergence of statistical differences.

In addition to the endpoints of our study, when the drugs were compared in terms of common side effects (headache, dizziness, and orthostatic hypotension), no significant difference was found, supporting previous studies [14,16-20]. Whereas, when retrograde ejaculation, another and more specific side effect of alpha blockers was considered, it was more frequently observed in patients taking silodosin [14,16-17,19-20]. It was observed that the number of our patients complaining of retrograde ejaculation in the tamsulosin group was found to be higher than in other studies. Except for the retrospective nature of the study, such a difference may have risen due to the cultural structure and shyness regarding sexual matters in our population.

## Conclusions

In the conservative treatment of low ureteral stones under 10 mm, MET appears to be a more effective method then WW. According to our results, no statistically significant superiority in terms of efficacy, duration, and side effects between tamsulosin and silodosin for distal ureteral stones was observed in the Turkish population.
